# Multi-Omics Elucidation of Edulinine’s Intervention Mechanism in Hypertensive Rats

**DOI:** 10.3390/cimb47120987

**Published:** 2025-11-26

**Authors:** Ling Tao, Junyou Jian, Tingting Chen, Xingjie Wu, Fei Jiang, Huaiju Ming, Dayao Han, Guangqiong Zhang, Lingyan Li, Shaobo Liu, Chunmao Yuan, Xiangchun Shen, Xiaojiang Hao

**Affiliations:** 1 State Key Laboratory of Discovery and Utilization of Functional Components in Traditional Chinese Medicine, School of Basic Medicine, Guizhou Medical University, No. 6 Ankang Avenue, Guian New District, Guiyang 561113, China; 2 The High Efficacy Application of Natural Medicinal Resources Engineering Center of Guizhou Province, The High Educational Key Laboratory of Guizhou Province for Natural Medicinal Pharmacology and Druggability, School of Pharmaceutical Sciences, Guizhou Medical University, No. 6 Ankang Avenue, Guian New District, Guiyang 561113, China; 3 State Key Laboratory of Phytochemistry and Plant Resources in West China, Kunming Institute of Botany, Chinese Academy of Sciences, Kunming 650201, China

**Keywords:** hypertension, edulinine, metabolomics, proteomics

## Abstract

Hypertension is a cardiovascular disorder characterized by sustained elevation of arterial blood pressure, in which vascular dysfunction serves as a key initiating factor leading to target organ injury. The indole alkaloid edulinine (Edu) represents a potential therapeutic agent for hypertension, although its specific mechanisms remain unclear. This study investigated the protective effects of Edu on vascular endothelial injury in N-⁠nitro-⁠L-⁠arginine-induced hypertensive rats using physiological, biochemical, and histopathological assessments. Through integrated proteomic and metabolomic analyses, we examined Edu’s effects on thoracic aortic tissue proteins and serum metabolic profiles to elucidate its molecular mechanisms. The results demonstrated that Edu exhibited superior antihypertensive efficacy compared to sodium nitroprusside and effectively ameliorated hypertension-induced left ventricular systolic dysfunction. Furthermore, proteomic analysis indicated that compared with the Model group, Edu showed significant intersections in the tricarboxylic acid cycle, fatty acid degradation, oxidative phosphorylation, and fatty acid elongation pathways. These pathways are of great significance to lipid metabolism and energy metabolism and are closely related to fatty acid elongation and myocardial contraction. In the fatty acid degradation pathway, the proteins up-regulated by Edu almost exactly correspond to those down-regulated by the Control group. Metabolomics analysis revealed that Edu exerts its antihypertensive effects primarily by regulating biological pathways involved in bile acid metabolism, fatty acid metabolism, and lipid metabolism. The integrated analysis of metabolomics and proteomics demonstrated that Edu markedly reduced the abnormal up-regulation of OXSM and MECR in hypertensive rats, suggesting that Edu may systematically regulate the balance of the fatty acid metabolic network by regulating the carbon chain initiation and elongation processes in fatty acid synthesis, as well as the key reductive reactions in mitochondrial β-oxidation. In summary, the potential mechanism of the protective effect and antihypertensive effect of Edu on the thoracic aorta of L-NNA-induced hypertensive rats may be inhibiting the up-regulation of OXSM and MECR expression, regulating the dynamic balance of fatty acid degradation and synthesis, and improving fatty acid metabolism disorders. These findings indicate that Edu holds substantial research value as a potential therapeutic candidate for hypertension.

## 1. Introduction

Worldwide, hypertension is the leading risk factor for cardiovascular disease and death, affecting about 300 million adults [1]. According to the Report on Cardiovascular Health and Diseases in China 2023, the prevalence rate among Chinese residents aged 18 and above is 31.6%, a sixfold increase compared to 1958. Moreover, the awareness rate, treatment rate and control rate are all relatively low [2]. It is generally agreed that the primary goal in the treatment of hypertension is to reduce overall blood pressure to below 140/90 mmHg, with a more optimal goal of 130/80 mmHg [3]. The high prevalence of hypertension not only significantly compromises patients’ quality of life but also imposes a substantial economic burden on the healthcare system. Vascular endothelial dysfunction serves as a critical link that not only initiates but also persists throughout the progression of hypertension. Despite more than a century of modern medical research, due to its complex and multifactorial pathogenesis, which is still not fully understood, posing significant challenges for effective prevention and treatment of hypertension [4]. In-depth investigation of the pathogenesis of vascular endothelial dysfunction and the development of targeted therapeutic drugs hold considerable scientific value for overcoming bottlenecks in hypertension prevention and treatment. Commonly used antihypertensive drugs—including diuretics, angiotensin II receptor blockers (ARBs), calcium channel blockers (CCBs), angiotensin-converting enzyme inhibitors (ACEIs), beta-blockers, and alpha-blockers—exhibit established therapeutic efficacy; however, they are associated with potential risks such as long-term toxicity, hepatorenal damage [5], and the development of drug resistance [6]. These limitations highlight the urgent need for developing novel antihypertensive agents that are both highly effective and safe. Natural compounds, particularly those derived from botanical sources, have attracted significant interest due to their multi-target regulatory effects and favorable safety profiles, offering distinct advantages for the long-term management of chronic conditions such as hypertension.

Edulinine (Edu), an indole alkaloid isolated from the root bark of *Orixa japonica* Thunb. (Rutaceae), has been shown to possess diverse biological activities, including sedative, analgesic, anti-inflammatory, centrally mediated inhibitory, and anticonvulsant effects [7,8]. These characteristics suggest that it may be involved in the pathological regulation of hypertension through multiple pathways. Under the condition of hypertension, chronic inflammation of the vascular wall induces VSMC proliferation and ECM remodeling by activating signaling pathways such as NF-κB [9], and the anti-inflammatory effect of Edu may reduce vascular injury by inhibiting such pathways. In addition, excessive excitation of the central nervous system, especially enhanced sympathetic activity, is an important driver of hypertension [10]. The central inhibitory effect of Edu may be involved in blood pressure regulation by regulating sympathetic output. At the same time, the structure of indoles is similar to some known alkaloids with vasodilation (such as reserpine derivatives) [11], suggesting that Edu may regulate vascular tension by affecting vascular endothelial function or ion channel activity. These mechanisms suggest that Edu may play a potential role in the key links of hypertension such as inflammation, central regulation and vascular function, and it is worth further exploring its specific targets and signaling pathways. However, there is no research report on the direct relationship between Edu and blood pressure regulation. Whether it can reduce blood pressure in hypertensive model animals, whether it has a protective effect on vascular endothelial injury, and what specific molecular mechanisms play a role remain to be answered.

The systematic evaluation of the antihypertensive potential and underlying mechanisms of Edu critically depends on modern omics technologies. Recent advances in proteomics and metabolomics have provided powerful approaches for elucidating complex pathophysiological mechanisms in hypertension. Proteomics enables high-throughput detection of changes in protein expression and post-translational modifications in tissues or cells, offering a comprehensive view of dysregulated protein networks under pathological conditions and facilitating the identification of key regulatory targets [12]. Metabolomics focuses on the dynamic alterations of small-molecule metabolites—such as amino acids, lipids, and organic acids—within biological systems. The analysis of metabolic profiles directly reflects the physiological and pathological states of organisms, revealing metabolic pathways involved in disease progression and responses to drug interventions [13]. Integrating both approaches enables a multidimensional interpretation along the “gene–protein–metabolite” axis, thereby overcoming the limitations of conventional single-target research. In addition to omics analyses, this study also employs conventional pathological and biochemical assessments—including hematoxylin and eosin (HE) staining for histopathological evaluation [14] and the detection of key biochemical indicators [15]—to comprehensively characterize the effects of Edu on hypertensive pathology and organ function.

Accordingly, this study employs a hypertensive rat model induced by N-nitro-L-arginine (L-NNA), which mimics hypertension arising from vascular endothelial dysfunction, closely recapitulating the pathological features of human essential hypertension [16]. Based on this model, we first evaluate the protective effects of Edu against vascular endothelial injury through physiological, biochemical, and histopathological assessments. We then apply proteomic and metabolomic approaches to investigate Edu-induced alterations in proteins from thoracic aortic tissues and serum metabolites, thereby elucidating the molecular mechanisms underlying its antihypertensive effects and providing a theoretical basis for potential clinical therapeutic strategies.

## 2. Method and Materials

### 2.1. Animal

Male Wistar rats weighing 200 ± 20 g (7 weeks) were provided by the Animal Center of Guizhou Medical University under the Production License No. SYXK (Qian, Guizhou, China) 2018-0001. All experimental procedures involving animals were approved by the Ethics Committee of Guizhou Medical University (Approval No. 1901109) and conducted in accordance with the Guide for the Care and Use of Laboratory Animals. The rats were housed under controlled environmental conditions with a temperature of 20–25 °C, humidity of 50–60%, and a 12 h/12 h light/dark cycle (light phase initiated at 07:00 a.m.). Throughout the study, they were provided with a standard rat pellet diet and had unrestricted access to water. Bedding was replaced twice weekly, and cages were sanitized every 3 days. After an acclimatization period of 7 days, all experiments were initiated.

### 2.2. Materials and Instruments

Edu was provided by Professor Xiaojiang Hao’s team at Guizhou Medical University (Batch No.: 20200115). The purity of Edulinine was determined to be ≥95% based on ^1^H NMR analysis [17]. Sodium Nitroprusside was purchased from Kaifeng Kangnuo Pharmaceutical Co., Ltd. (Kaifeng, China, Batch No.: 154154). L-NNA was obtained from Shanghai Macklin Biochemical Technology Co., Ltd. (Shanghai, China, Product Code: C11466030). Standard rodent diet (16% protein, 4% fat, and 60% carbohydrate) was supplied by Chongqing Tengxin Biotechnology Co., Ltd. (Chongqing, China, Batch No.: 2017-1021). Instrumentation included a Thermo Scientific QE Orbitrap High-Resolution Mass Spectrometer (Thermo Fisher Scientific, Waltham, MA, USA) coupled with a Vanquish Ultra-High-Performance Liquid Chromatography system (Thermo Fisher Scientific, Waltham, MA, USA).

### 2.3. Animal Models and Drug Administration

The hypertensive rat model with vascular endothelial injury was induced through daily intraperitoneal injection of L-NNA, dissolved in physiological saline at 1.5 mg/mL, administered at a dose of 2.25 mg/100 g·d for 4 weeks. Systolic blood pressure (SBP), diastolic blood pressure (DBP), and mean arterial pressure (MAP) were monitored weekly using a non-invasive BP6 tail-cuff system. After 4 weeks, rats exhibiting SBP > 140 mmHg and DBP > 90 mmHg were selected and randomly assigned to the Model group, positive Control group (sodium nitroprusside, 1 mg/kg/d), or Edu treatment group (6 mg/kg/d), with six rats per group. One hour after the daily L-NNA administration, sodium nitroprusside or Edu was injected intraperitoneally; the Model and Control groups received an equivalent volume of saline. Prior to measurements, all animals were acclimatized through training sessions. Rats were held at 37 °C for 10–15 min to make tail artery pulsations detectable, minimizing stress-induced fluctuations. Tail artery blood pressure was then recorded at 15, 30, 45, 60, and 120 min following a single administration. All treatments were maintained for 2 weeks, with blood pressure measurements taken once per week.

Body weight was recorded every 7 days throughout the study. Cardiac index was assessed using a small animal ultrasound system at three time points: before model induction, after 4 weeks of modeling (prior to drug administration), and after 2 weeks of treatment, to evaluate the impact of Edu on cardiac function in L-NNA-induced hypertensive rats. For serum analysis, blood samples were collected from the abdominal aorta, centrifuged at 3000 rpm for 10 min, and the serum supernatant was isolated. Levels of triglycerides (TG), high-density lipoprotein (HDL), low-density lipoprotein (LDL), glucose (GLU), and glycated serum protein (GSP) were quantified using a fully automated biochemical analyzer, with six rats per group.

### 2.4. Histopathological Staining Protocols

The thoracic aorta samples from rats in each group were fixed in 4% paraformaldehyde, embedded in paraffin, sectioned, dewaxed, and rehydrated. Histological examination of tissue morphology was conducted using 4 μm thick sections stained with hematoxylin and eosin (HE). The sections were mounted with neutral balsam and observed under a light microscope for image acquisition. For Masson staining, paraffin sections of rat thoracic aorta were dewaxed, subjected to Masson staining, permeabilized, and mounted. The pathological changes in the tissues were observed under a microscope and images were captured. For Oil Red O staining, the thoracic aorta samples from each group were fixed in 4% paraformaldehyde and dehydrated in isopropanol. The samples were horizontally placed in embedding molds and dried in a 60 °C incubator for 2 h. The dried vessels were immersed in a 0.5% Oil Red O staining solution for 30 min. After staining, differentiation was performed with 60% isopropanol for several seconds until the background became clear, and the reaction was terminated by PBS rinsing. Finally, the nuclei were counterstained with hematoxylin, and blueing was achieved with PBS. The sections were dehydrated through a graded ethanol series (70%, 80%, 95%, and 100% for 2 min each), cleared in xylene, mounted with neutral balsam, and observed under an optical microscope to evaluate the degree of lipid deposition in the vessel wall.

### 2.5. Serum Metabolomics Analysis

A 50 μL aliquot of abdominal aorta serum from each group of rats was mixed with 300 μL of a 20% acetonitrile-methanol internal standard extraction solution. The mixture was vortex-mixed for 3 min and centrifuged at 4 °C and 12,000 rpm for 10 min. Subsequently, 200 μL of the supernatant was collected and placed in a −20 °C freezer for 30 min. This was followed by another centrifugation step under the same conditions (4 °C, 12,000 rpm) for 3 min, after which 180 μL of the supernatant was taken for instrumental analysis. Chromatographic separation was performed using a Waters ACQUITY Premier HSS T3 column (1.8 μm, 2.1 mm × 100 mm). Mobile phase A was a 0.1% formic acid aqueous solution, and mobile phase B was a 0.1% formic acid acetonitrile solution. The column temperature was set at 40 °C, the flow rate was 0.4 mL/min, and the injection volume was 4 μL. The raw mass spectrometry data were converted to mzXML format and preprocessed using XCMS software (v. 3.7.1) for peak extraction, alignment, retention time correction, filtering, filling, and recalibration. Compound identification was subsequently conducted, screening for substances with a composite score above 0.5 and a quality control (QC) sample coefficient of variation (CV) less than 0.3. Data from positive and negative ion modes were then merged (prioritizing substances with the highest identification level and the smallest CV value) to obtain the relevant analysis files.

### 2.6. Proteomics Analysis

Protein quantitative analysis was performed on thoracic aortic tissues from each group of rats, with three samples randomly selected per group. After protein extraction, the sample concentrations were determined using the BCA method. The samples subsequently underwent reductive alkylation treatment, followed by tryptic digestion to prepare peptide samples. The resulting peptides were desalted and quantified, then analyzed by ultra-high-performance liquid chromatography tandem mass spectrometry (DIA) technology. Qualitative and quantitative analyses were conducted using Spectronaut™ software (v. 19) based on a spectral library. Further bioinformatics analysis was performed, including the generation of volcano plots to screen for differentially expressed proteins in comparison groups with *t*-test *p*-values. The screened differentially expressed proteins were subsequently imported into the KOBSA website for Gene Ontology (GO) and Kyoto Encyclopedia of Genes and Genomes (KEGG) analyses.

### 2.7. Molecular Docking

Molecular docking was performed using Schrödinger’s Maestro software (Release 2025-1) to evaluate the binding affinity of Edu to Acads, Acadm, Acat1, Acadl, Hadh, Hadha, and Hadhb. The protein structures of Acads (PDB ID: 7YOA), Acadm (PDB ID: 1t9g), Acat1 (PDB ID: 6P2J), Hadh (PDB ID: 3RQS) and Hadha (PDB ID: 5ZQZ) were downloaded from PDB database (https://www.rcsb.org/). The protein structures of Acadl and Hadhb predicted by AIphafold were downloaded from Uniprot database (https://www.uniprot.org/) and imported into Maestro. Structural corrections, including dehydration, hydrogen bond optimization, and energy minimization, were conducted using the Protein Preparation Wizard module. For proteins lacking ligand-bound structures, potential active sites were predicted using the SiteMap module. A docking grid (generated as glide-grid_1.zip) was defined centered on either the reference ligand or predicted active sites via the Receptor Grid Generation module. The 2D structure of L-NNA was obtained from PubChem, and its optimized 3D conformation was generated using the LigPrep module (output stored in ligprep_1 folder). Preprocessed proteins and ligands were subjected to molecular docking in the Ligand Docking module with Extra Precision (XP) mode. Lower docking scores indicated stronger binding stability. Protein-ligand interactions were analyzed using PLIP v2.3.0 (https://plip-tool.biotec.tu-dresden.de/plip-web/plip/index, accessed on 8 January 2025) and resulting pse files were further visualized with PyMOL (v. 3.1) and Discovery Studio Visualizer (v. 2025).

### 2.8. Statistics

GraphPad Prism software (version 9.5.0) was used for data analysis. Measurement data were expressed as mean ± standard deviation. The *t*-test was used to compare means between two groups. One-way ANOVA was used to compare means among three groups. A value of *p* < 0.05 was considered statistically significant.

## 3. Results

### 3.1. Antihypertensive Effect of Edu on L-NNA-Induced Hypertensive Rats

**Figure 1:**
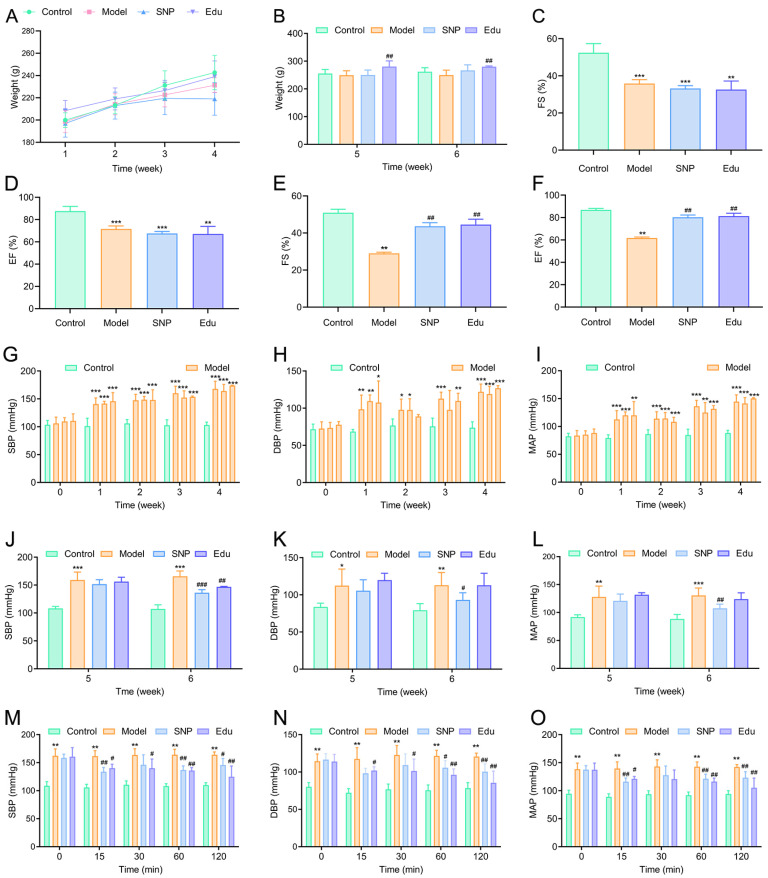
Effects of Edu on body weight, cardiac function, and blood pressure in hypertensive rats. (**A**,**B**) Changes in body weight over the first 4 weeks after treatment with SNP or Edu (**A**), and body weight at weeks 5 and 6 post-treatment (**B**). (**C**,**D**) Fractional shortening (FS) and left ventricular ejection fraction (EF) measured by non-invasive ultrasound before treatment. (**E**,**F**) FS and EF measured 2 weeks after treatment. (**G**–**I**) Changes in systolic blood pressure (SBP), diastolic blood pressure (DBP), and mean arterial pressure (MAP) before treatment. (**J**–**L**) Changes in SBP, DBP, and MAP 2 weeks after treatment. (**M**–**O**) Changes in SBP, DBP, and MAP at 15, 30, 45, 60, and 120 min after a single administration of SNP or Edu. Control (untreated), Model (L-NNA-induced hypertension), SNP (model rats administered with 1 mg/kg/day SNP), Edu (model rats administered with 6 mg/kg/day Edu). Data are presented as mean ± SD (n = 6). *, **, and *** indicated significantly different to Control group with *p* < 0.05, *p* < 0.01, and *p* < 0.001, respectively; #, ##, and ### indicated significantly different to Model group with *p* < 0.05, *p* < 0.01, and *p* < 0.001, respectively.

Quantitative assessment of left ventricular systolic function primarily relies on measurements of left ventricular chamber dimensions and volume changes. Clinically, indicators such as ejection fraction (EF) and fractional shortening (FS) are commonly used to evaluate left ventricular function [18]. The body weight elevations of rats were observed at six-week-injection, with the Edu group maintaining relatively stable body weight (Figure 1A,B). Compared with the Control group, the EF and FS values of the L-NNA-induced hypertensive rat model were significantly reduced (Figure 1C–F), indicating that this modeling method effectively induced impaired left ventricular systolic function in rats. After Edu treatment, both EF and FS indicators showed significant improvement, demonstrating that Edu has a notable therapeutic effect on the left ventricular systolic dysfunction induced by L-NNA-induced hypertension. Regarding body weight changes, no significant differences were observed among the Model groups during the first 4 weeks.

Hypertension is a clinical syndrome characterized by persistently elevated systemic arterial blood pressure (including systolic and/or diastolic pressure), typically defined as systolic blood pressure ≥ 130 mmHg and/or diastolic blood pressure ≥ 80 mmHg, and is often accompanied by functional or structural impairments in vital organs such as the heart, brain, and kidneys [19,20]. In the field of pharmacological research, L-NNA is a commonly used inducer for constructing models of hypertension and thoracoabdominal aortic vascular pathological injury [16]. In this study, rats subjected to intraperitoneal injection of L-NNA exhibited a gradual increase in blood pressure after one week. By the fourth week, 90% of the rats had systolic blood pressure (SBP) exceeding 140 mmHg and diastolic blood pressure (DBP) exceeding 90 mmHg. In the single-dose administration experiment, both the sodium nitroprusside (SNP) group and the Edu group showed a significant reduction in systolic blood pressure compared to the Model group (Figure 1G–L). Regarding diastolic blood pressure, the SNP group exhibited a statistically significant decrease at 60 and 120 min after administration, while the Edu group showed statistically significant reductions at 15, 30, 60, and 120 min after administration (Figure 1M–O).

### 3.2. Ameliorative Effects of Edu on Abnormal Biochemical Indicators and Histopathological Injury

**Figure 2:**
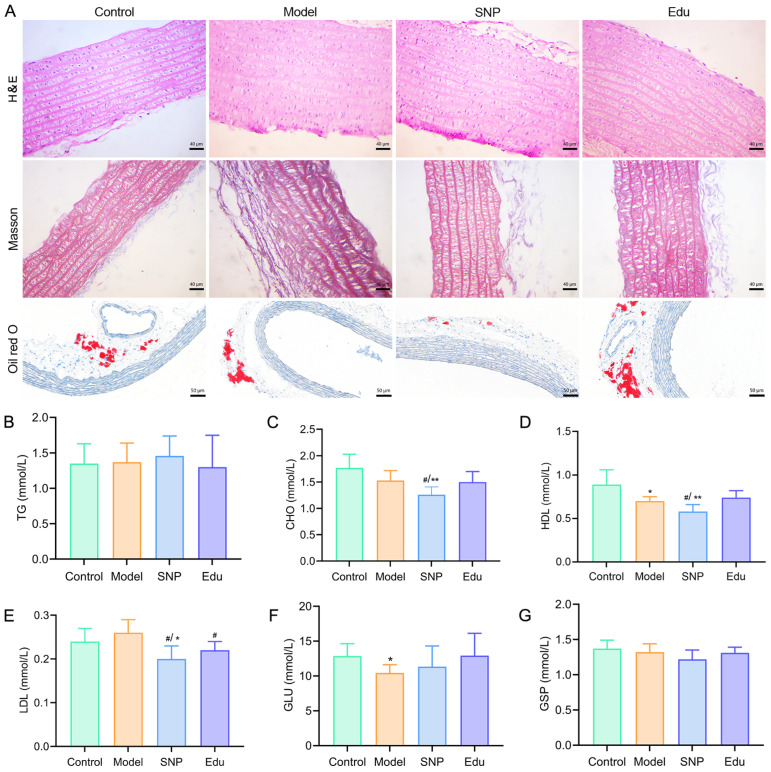
Improvement effects of Edu on biochemical indices and histopathology in rats. (**A**) Hematoxylin and eosin (H&E) staining, Masson’s trichrome staining, and Oil Red O staining of the thoracic aorta from each group of rats after two weeks of treatment with SNP or Edu. (**B**–**G**) Serum levels of triglycerides (TG), cholesterol (CHO), high-density lipoprotein cholesterol (HDL), low-density lipoprotein cholesterol (LDL), glucose (GLU), and glycated serum protein (GSP) measured using an automatic biochemical analyzer (AU5810, Bechman, CA, USA) after two weeks of treatment with SNP or Edu. Control (untreated), Model (L-NNA-induced hypertension), SNP (model rats administered with 1 mg/kg/day SNP), Edu (model rats administered with 6 mg/kg/day Edu). Data are expressed as mean ± SD (n = 6). * and ** indicated significantly different to Control group with *p* < 0.05 and *p* < 0.01, respectively; # indicated significantly different to Model group with *p* < 0.05.

Pathological section results indicated that in the Control group, the aortic vascular intima exhibited focal hyperplasia, the media showed focal smooth muscle-like degeneration and vacuolar degeneration, and the adventitia presented focal fibrinoid degeneration. In the Model group, the aortic intima displayed focal hyperplasia with extracellular lipids, the media exhibited focal smooth muscle hyperplasia, hyaline degeneration of smooth muscle, and vacuolar degeneration, and the adventitia demonstrated fibrinoid degeneration. In the SNP group, the intima showed focal hyperplasia with extracellular lipids and scattered inflammatory cells adherent to the intima. Similarly, the Edu group presented focal intimal hyperplasia, extracellular lipids, and scattered inflammatory cells attached to the intima (Figure 2A). Masson staining results revealed that in the Model group, the thoracic aorta of rats exhibited disorganized and disrupted elastic fiber structures along with a significant increase in collagen content. In contrast, both the SNP and Edu groups showed a significant reduction in the blue-stained area of collagen fibers compared to the Model group, with relatively intact elastic fiber structures in the vascular wall and only minor localized fractures (Figure 2A). Additionally, smooth muscle cells were arranged more orderly than those in the Model group, and clear blue–purple staining of cell nuclei was visible. Oil Red O staining showed that the vascular tissue structures remained intact across all groups, with no significant lipid droplets observed (Figure 2A), indicating the absence of notable lipid deposition in the vascular walls of rats in any group.

Blood lipid and glucose profiles at the end of the 6th week revealed that compared to the Control group, the Model group showed increased levels of triglyceride (TG), total cholesterol (CHO), and low-density lipoprotein cholesterol (LDL), although the differences were not statistically significant (*p* > 0.05). In contrast, high-density lipoprotein cholesterol (HDL) levels were significantly decreased (*p* < 0.05), suggesting early dysregulation of the lipid profile in the Model group. In the Edu intervention group, TG, CHO, and HDL levels did not differ significantly from those in the Control group (*p* > 0.05), indicating that Edu had no substantial impact on normal lipid metabolism. Compared to the Model group, both the sodium nitroprusside (SNP) group and the Edu group exhibited significantly reduced LDL levels (*p* < 0.05). However, the SNP group’s LDL and HDL levels were also significantly lower than those of the Control group (*p* < 0.05) (Figure 2B–E), implying that SNP may influence normal lipid metabolism beyond the pathological context, whereas Edu’s LDL-lowering effect appeared more selective toward the hypertension-induced dyslipidemia.

Blood glucose metabolism evaluation revealed that in the hypertensive animal model, glucose levels may be influenced by vascular endothelial dysfunction, oxidative stress, inflammatory responses, or modeling drug interactions, leading to acute or short-term fluctuations [21,22]. This study measured glucose (GLU) to determine whether model construction or drug intervention affected metabolic stability. The results showed that compared with the Control group, the Model group exhibited a significant decrease in GLU levels (*p* < 0.05), suggesting that the modeling process may be accompanied by abnormal glucose regulation. In contrast, the Edu intervention group showed no statistically significant differences in GLU levels compared to either the Model group or the Control group (*p* > 0.05). Combined with the absence of significant changes in glycated serum protein (GSP) levels across all groups (Figure 2F–G), these findings further confirm that Edu, at the current dosage and experimental duration, did not induce elevation or reduction in blood glucose, demonstrating no adverse effects on glucose metabolism and indicating favorable metabolic safety.

### 3.3. Serum Metabolomics Investigation of the Regulatory Mechanism of Edu in Improving Metabolic Disorders in Hypertensive Rats

**Figure 3:**
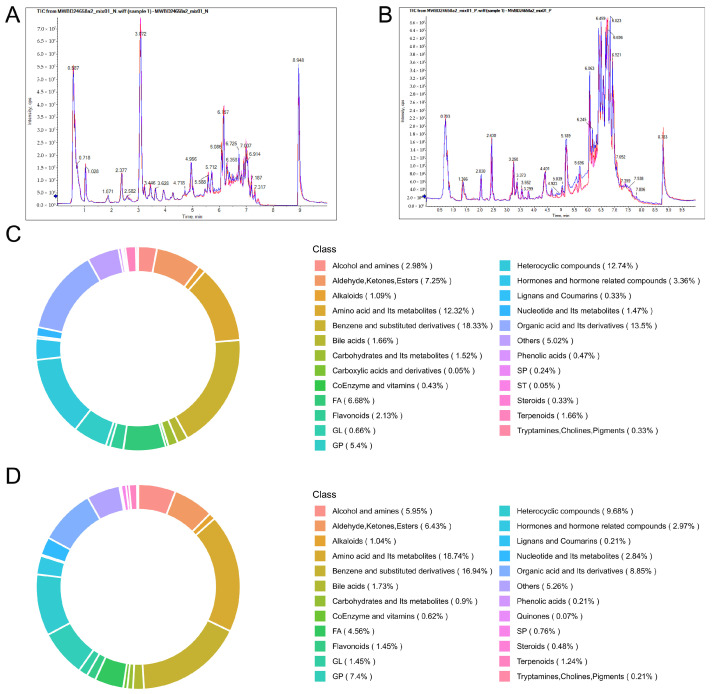
Mass spectrometry total ion current (TIC) overlap and metabolite classification of quality control (QC) samples. (**A**) Overlaid TIC of QC samples in negative ion mode. (**B**) Overlaid TIC of QC samples in positive ion mode. (**C**) Ring chart of metabolite category composition in the Model group. (**D**) Ring chart of metabolite category composition in the Edu group. Model (L-NNA-induced hypertension), Edu (model rats administered with 6 mg/kg/day Edu).

To investigate the changes induced by Edu in the treatment of hypertension at the systemic metabolic level, this study employed ultra-performance liquid chromatography coupled with electrospray ionization tandem mass spectrometry (UPLC-Q-Exactive Orbitrap-HRMS) to analyze alterations in serum components of hypertensive rats following intraperitoneal administration of Edu. The superimposed total ion current (TIC) chromatograms of quality control (QC) samples exhibited high overlap in metabolic detection profiles (Figure 3A,B), demonstrating consistent retention times and peak intensities across replicate analyses and confirming robust signal stability of the mass spectrometry system over time. Metabolite classification results (Figure 3C,D) delineated the compositional characteristics with the predominant classes in positive ion mode being benzene and its derivatives (18.33%), organic acids (13.5%), and heterocyclic compounds (12.74%), collectively representing 44.57% of the total metabolites, while in negative ion mode, amino acids and their metabolites (18.74%) constituted the most abundant category, followed by organic acids (8.85%) and benzene derivatives (8.85%) at equal proportions, with heterocyclic compounds (9.68%) ranking third.

**Figure 4:**
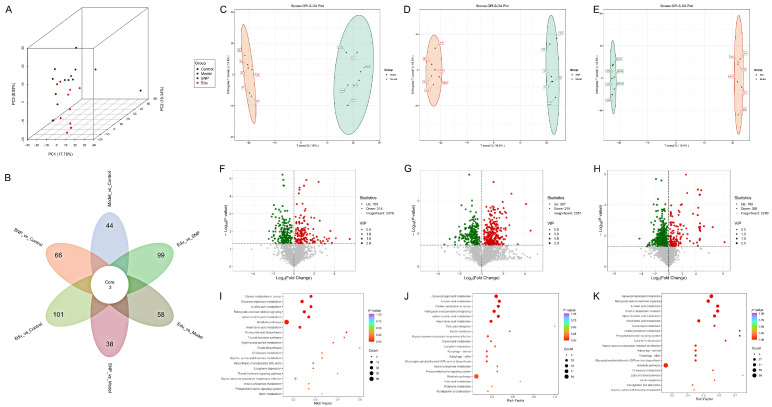
Metabolomics analysis. (**A**) Principal component analysis (PCA) of differential metabolites. (**B**) Venn diagram of differences among each group. (**C**) OPLS-DA score plot of Model vs. Control. (**D**) OPLS-DA score plot of SNP vs. Model. (**E**) OPLS-DA score plot of Edu vs. Model. (**F**) Volcano diagram of differential metabolites between Model vs. Control. (**G**) Volcano diagram of differential metabolites between SNP vs. Model. (**H**) Volcano diagram of differential metabolites between Edu vs. Model. (**I**) The differential metabolite pathway enrichment diagram of Model vs. Control. (**J**) The differential metabolite pathway enrichment diagram of SNP vs. Model. (**K**) The differential metabolite pathway enrichment diagram of Edu vs. Model. Control (untreated), Model (L-NNA-induced hypertension), SNP (model rats administered with 1 mg/kg/day SNP), Edu (model rats administered with 6 mg/kg/day Edu).

We employed both principal component analysis (PCA) and orthogonal partial least squares-discriminant analysis (OPLS-DA) to elucidate differences among the various groups. The PCA score plot demonstrated that the inter-group differences and intra-group variations met the required criteria (Figure 4A). Venn diagram analysis of the differential metabolites affected by Edu and SNP treatments revealed three common differential metabolites shared between the two treatment groups (Figure 4B). Furthermore, OPLS-DA analysis revealed that the metabolic profiles of the Model group and the Edu group formed distinct clusters, located in four separate regions of the score plot, suggesting that Edu has the potential to ameliorate metabolic abnormalities or serve as a distinguishing indicator at the metabolic level (Figure 4C–E). Differential metabolites were identified based on the criteria of fold change (FC > 1 or FC < −1), *p*-value < 0.05, and variable importance in projection (VIP) > 1, and volcano plots of these differential metabolites were generated. Compared with the normal Control group, the Model group exhibited significant changes in 379 metabolites, with 165 metabolites up-regulated and 214 down-regulated. In the Edu treatment group, 505 metabolites showed significant changes compared to the Model group, including 150 up-regulated and 355 down-regulated metabolites. The SNP treatment group also showed significant changes in 505 metabolites, with 287 up-regulated and 218 down-regulated (Figure 4F–H). Further analysis showed that compared with the Control group, the Model group was significantly up-regulated; at the same time, compared with the Model group, the metabolites significantly down-regulated in the Edu group were mainly NBD-stearoyl-2-arachidonoyl-sn-glycerol, Cys-Tyr, glycocholic acid, 1-octadecanoyl-2- (9Z-octadecenoyl) -sn-glycero-3-phosphate, nitroarginine, idebenone (Supplementary Tables S1 and S2). The above differentially expressed metabolites are involved in biological pathways such as fatty acid metabolism, amino acid metabolism, lipid metabolism, and primary bile acid biosynthesis in the body. The differential metabolites were imported into the KEGG database for pathway analysis. Metabolic pathways with a *p*-value < 0.05 and an impact value > 0.1 were screened as potential pathways, and the top 20 pathways with the smallest *p*-values were selected to construct KEGG bubble plots (Figure 4I–K). Compared with the normal Control group, hypertensive rats showed significant alterations in pathways such as glycerophospholipid metabolism, linoleic acid metabolism, retrograde endocannabinoid signaling, linolenic acid metabolism, arachidonic acid metabolism, and metabolic pathways. After Edu treatment, notable changes were observed in arachidonic acid metabolism, retrograde endocannabinoid signaling, and metabolic pathways, with the most significant alteration occurring in the metabolic pathways.

### 3.4. Proteomics Analysis

**Figure 5:**
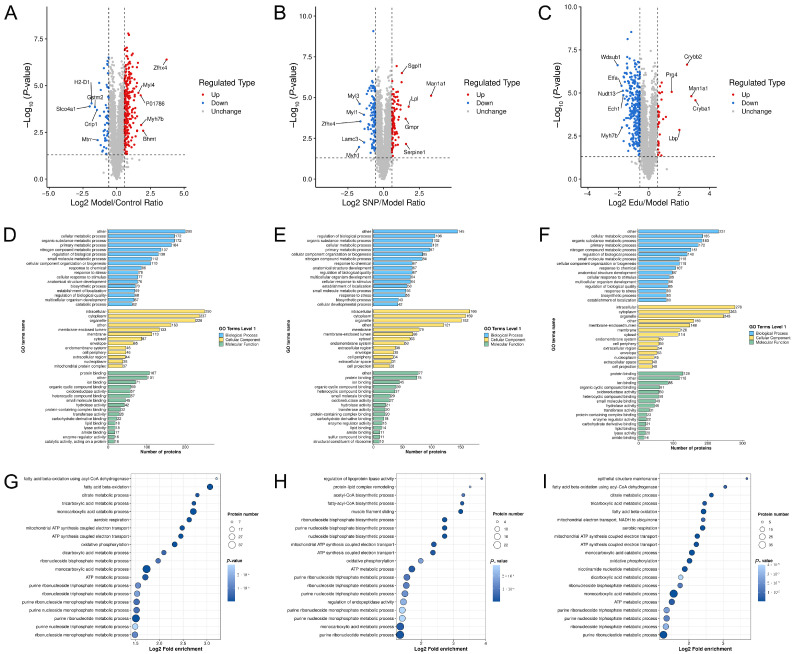
Proteomic analysis of the thoracic aorta. (**A**) Volcano plot of differentially expressed proteins between the Model group and the Control group. (**B**) Volcano plot of differentially expressed proteins between the SNP group and the Model group. (**C**) Volcano plot of differentially expressed proteins between the Edu group and the Model group. (**D**) GO analysis of differentially expressed proteins between the Model group and the Control group. (**E**) GO analysis of differentially expressed proteins between the SNP group and the Model group. (**F**) GO analysis of differentially expressed proteins between the Edu group and the Model group. (**G**) KEGG pathway analysis of differentially expressed proteins between the Model group and the Control group. (**H**) KEGG pathway analysis of differentially expressed proteins between the SNP group and the Model group. (**I**) KEGG pathway analysis of differentially expressed proteins between the Edu group and the Model group. Control (untreated), Model (L-NNA-induced hypertension), SNP (model rats administered with 1 mg/kg/day SNP), Edu (model rats administered with 6 mg/kg/day Edu).

To examine changes in differential proteins, criteria were set at a *p*-value < 0.05 and |FC| ≥ 1.5. The results demonstrated that compared to the normal Control group, the Model group exhibited significant alterations in 292 proteins, with 245 proteins up-regulated and 47 down-regulated. Compared to the Model group, the Edu treatment group showed significant changes in 320 proteins, including 285 down-regulated and 35 up-regulated. In contrast, the SNP positive Control group displayed significant alterations in 188 proteins, with 86 up-regulated and 102 down-regulated (Figure 5A–C).

GO annotation encompasses three categories biological process (BP), cellular component (CC), and molecular function (MF) which elucidate the biological functions of proteins from multiple perspectives. The GO results indicated that BP was primarily associated with metabolic process and crosslinking, CC demonstrated substantial associations with intracellular mitochondrial protein complexes, and MF was related to oxidoreductase activity (Figure 5D–F). KEGG pathway analysis of the differentially expressed proteins revealed that the bubble map for Model vs. Control co-regulation showed protein enrichment in fatty acid degradation, TCA cycle, pyruvate metabolism, oxidative phosphorylation, among others, which held significant implications for lipid metabolism and energy metabolism and was closely related to cardiac muscle contraction. The Edu vs. Model group exhibited significant intersections in the TCA cycle, fatty acid degradation, oxidative phosphorylation pathways. In contrast, the single nucleotide polymorphism group showed limited involvement, only in the TCA cycle and fatty acid degradation pathways. Notably, in the fatty acid degradation pathway, the proteins up-regulated by Edu corresponded almost entirely to those down-regulated in the Control group, and this number was significantly greater than that in the SNP group (Figure 5G–I).

### 3.5. Molecular Docking Results

**Figure 6:**
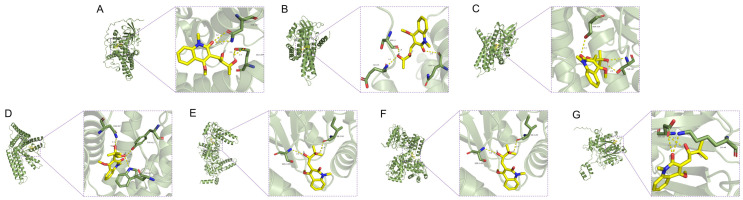
Molecular models of Edu binding to their predicted key protein targets. (**A**–**G**) Molecular docking of Edu with Acads (**A**), Acadm (**B**), Acat1 (**C**), Acadl (**D**), Hadh (**E**), Hadha (**F**), and Hadhb (**G**), respectively. Control (untreated), Model (L-NNA-induced hypertension), Edu (model rats administered with 6 mg/kg/day Edu).

To thoroughly investigate the effect of Edu on key enzymes involved in fatty acid degradation, molecular docking technology was employed to simulate the binding affinity between Edu and critical fatty acid degradation enzymes, including Acads, Acadm, Acat1, Acadl, Hadh, Hadha, and Hadhb. The results demonstrated that the binding energies of Edu with Acads, Acadm, Acat1, Acadl, Hadh, Hadha, and Hadhb were −8.4, −8.1, −7.3, −6.3, −7.1, −6.9, and −6.6 kcal/mol, respectively (Figure 6). These findings indicate that Edu exhibits strong binding capabilities to key enzymes in fatty acid degradation, suggesting that Edu may delay the progression of hypertension by interfering with the fatty acid degradation pathway.

### 3.6. Integrated Analysis of Metabolomics and Proteomics

#### 3.6.1. Ipathway Analysis

**Figure 7:**
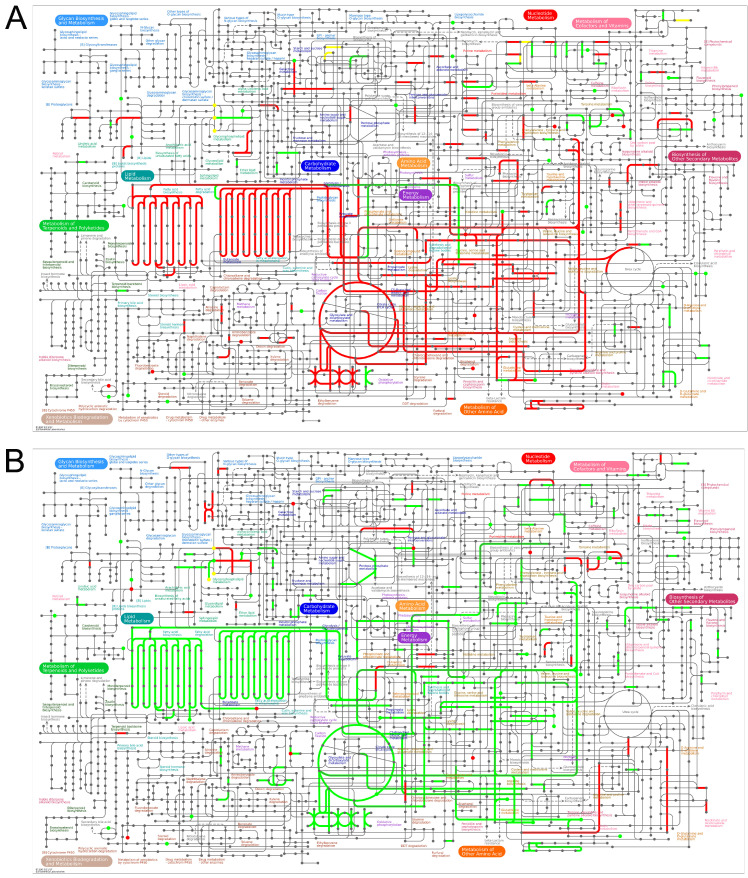
Proteomics and metabolomic correlation analysis. (**A**) The iPath pathway of differential metabolites and differential genes in the Model group and Control group (**B**) The iPath pathway of differential metabolites and differential genes in the Edu group and Model group. Nodes represent biochemical molecules, and lines represent biochemical reactions. Different colors in the pathway indicate the differential expression of proteins and metabolites. Red indicates up-regulation, green indicates down-regulation, and yellow indicates both up-regulation and down-regulation. Control (untreated), Model (L-NNA-induced hypertension), Edu (model rats administered with 6 mg/kg/day Edu).

There exists a close interaction and regulatory relationship between proteins and metabolites. To further elucidate the mechanism of Edu in hypertension treatment, we conducted an integrated analysis of proteins data and metabolomics data. For the proteomic data, differentially expressed proteins were screened based on the criteria of FC > 1.3 or FC < 1/1.3 with a *p*-value < 0.05. In the metabolomic analysis, the basic criteria were set as |log2FC| > 0 and *p* < 0.05, with VIP > 1 introduced as an additional filter when data availability permitted. To further investigate the co-regulatory relationships between differential proteins and differential metabolites at the biological pathway level and their alterations under hypertensive pathological conditions and Edu intervention, we utilized iPath software (v. 3) to systematically visualize the biochemical pathways co-enriched by both differential metabolites and differential proteins. In this visualization, nodes represent various biochemical molecules (e.g., metabolic intermediates, enzymes), and connecting lines indicate the biochemical reactions or metabolic fluxes in which they participate, thereby intuitively revealing the network interactions among different metabolic and signaling pathways. The iPath pathway visualization results demonstrated that, compared to the Control group, the Model group exhibited significant alterations in multiple core metabolic and biosynthetic pathways, including lipid metabolism, carbohydrate metabolism, amino acid metabolism, energy metabolism, nucleotide metabolism, and biosynthesis of secondary metabolites (Figure 7A), suggesting that the hypertensive pathological state is accompanied by multi-pathway metabolic dysregulation. On the other hand, the comparison between the Edu intervention group and the Model group revealed that Edu primarily induced significant differences in lipid metabolism, carbohydrate metabolism, amino acid metabolism, and energy metabolism (Figure 7B), indicating that Edu may exert its antihypertensive and metabolic protective effects by modulating these metabolic networks.

#### 3.6.2. Kegg Pathway Enrichment Analysis

**Figure 8:**
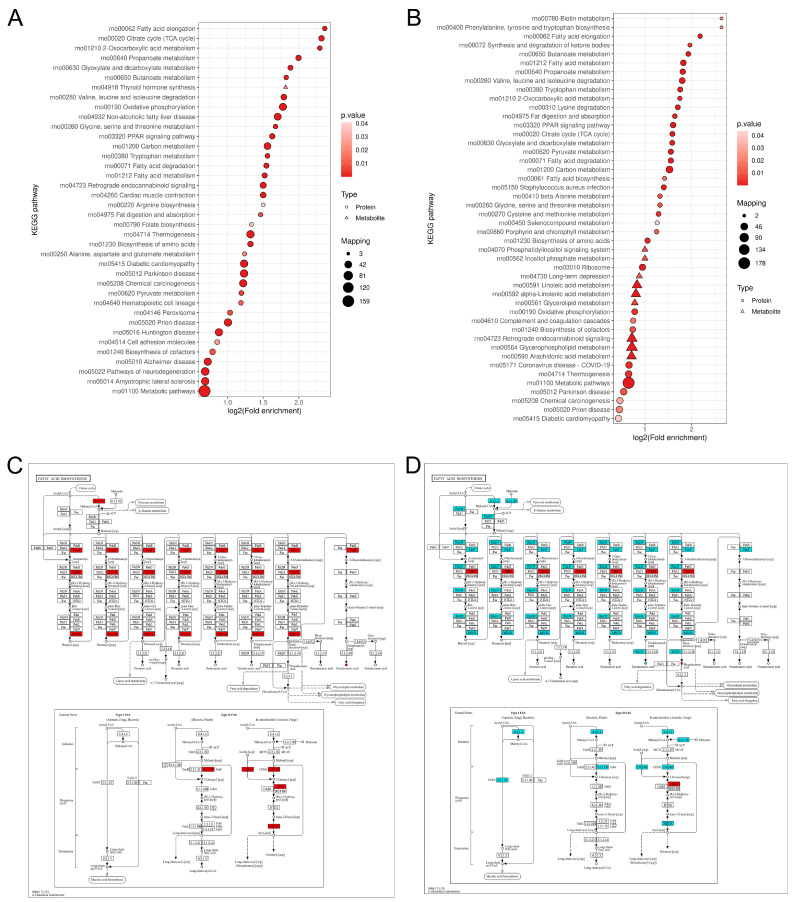
Correlation analysis between serum metabolomics and proteomics of the thoracic aorta. (**A**) Bubble plot of KEGG pathway enrichment analysis of differentially expressed proteins and metabolites between the Model group and the Control group. (**B**) Bubble plot of KEGG pathway enrichment analysis of differentially expressed proteins and metabolites between the Edu group and the Model group. (**C**) Visualization of KEGG pathways between the Model group and the Control group. (**D**) Visualization of KEGG pathways between the Edu group and the Model group. The rectangular frame represents the enzyme or key intermediate, the circle represents the metabolite, the black solid line arrow indicates the main reaction step, and the dotted line arrow indicates the associated metabolic pathway or secondary process. Different colors in the pathway expressed the differential expression of proteins and metabolites. Red indicated up-regulation and blue indicated down-regulation. Control (untreated), Model (L-NNA-induced hypertension), Edu (model rats administered with 6 mg/kg/day Edu).

We employed Fisher’s exact test to perform KEGG pathway enrichment analysis on both differentially expressed proteins and metabolites, with a significance threshold set at *p* < 0.05 for identifying significantly enriched pathways. The enrichment results are visualized in the corresponding figures, where the horizontal axis represents the log2-transformed enrichment factor, and the vertical axis displays the descriptions of the KEGG pathways. Triangles denote metabolites, while circles represent proteins. The color of the points indicates the significance of enrichment, with red indicating stronger enrichment significance. The size of the points corresponds to the number of metabolites or proteins involved in the pathway. The results demonstrated that in the comparison between the Model and Control groups, fatty acid metabolism-related pathways (such as fatty acid elongation, fatty acid biosynthesis, and fatty acid degradation) were significantly enriched, with multiple metabolism-related entries ranking among the top five most significant pathways in the metabolic network. In the comparison between the Edu and Model groups, fatty acid metabolism exhibited a high enrichment factor (Figure 8A,B). These findings suggest that fatty acid metabolism is one of the primary metabolic pathways involved in both the pathological state of hypertension and the response to Edu intervention.

According to the KEGG pathway analysis results, combined with the visual analysis of differential proteins and metabolites (circles represent metabolites, squares represent proteins; red indicates up-regulation, green indicates down-regulation, and yellow indicates the presence of multiple expression patterns within the same square), the results demonstrated that in the comparison between the Model and Control groups, the fatty acid biosynthesis pathway was one of the significantly enriched metabolic pathways in the differential expression analysis, with 28 proteins significantly up-regulated and no down-regulated proteins in this pathway. In the comparison between the Edu and Model groups, 66 proteins in this pathway still exhibited significant changes, including 8 up-regulated and 58 down-regulated proteins (Figure 8C,D). Notably, the expression levels of 3-oxoacyl-ACP synthase (OXSM), a key enzyme catalyzing the initiation and elongation of fatty acid carbon chains [23], and mitochondrial trans-2-enoyl-CoA reductase (MECR), which mediates a critical reductive step in mitochondrial β-oxidation [24], were significantly up-regulated in the Model group compared to the Control group. In contrast, Edu treatment markedly downregulated the expression of both OXSM and MECR.

## 4. Discussion

Hypertension serves as a significant pathogenic factor for various cardiovascular diseases. Prolonged hypertension can lead to cardiac and vascular damage, inducing myocardial hypertrophy and vascular wall thickening [1,25]. In this study, after four weeks of continuous intraperitoneal injection of L-NNA, rat blood pressure elevated to hypertensive levels. Upon continuing L-NNA injection until the sixth week, the Model group maintained persistently high blood pressure alongside pathological alterations in arterial vessels, manifesting as focal intimal hyperplasia, extracellular lipid deposition, focal medial smooth muscle hyperplasia, hyaline degeneration, vacuolar degeneration, and adventitial fibrinoid degeneration. These characteristics are largely consistent with existing research findings [26,27]. Additionally, reductions in left ventricular EF and FS were observed, aligning with features reported in previous studies of hypertension and cardiac remodeling induced by L-arginine analogs [28], further confirming the stability of the present model. The single-dose administration experiment showed that the Edu group had the same effect as the SNP group in reducing SBP, but it was more significant in reducing DBP, and the effect lasted longer. Compared with the SNP group, Edu not only had a similar or earlier onset time, but also maintained its antihypertensive effect to 120 min, suggesting that Edu had an acute antihypertensive ability comparable to that of SNP, and showed a more lasting diastolic blood pressure regulation. In addition, studies have found that Edu can significantly reverse the abnormal reduction in left ventricular EF and FS, confirming its protective effect on hypertensive cardiac dysfunction.

Hypertensive patients has received extensive attention of accompanied by metabolic abnormalities such as obesity, abnormal blood glucose [29], dyslipidemia [30], elevated uric acid [31]; metabolic abnormalities have become one of the main reasons for the increase in the prevalence of hypertension, occupying the dominant position in the pathogenesis of hypertension [32], and accompanied by the whole process of hypertension, increasing the damage to the target organs [33]. Lectin-like oxidized low density lipoprotein receptor 1 (LOX-1) is a specific receptor of oxLDL [34]. When hypertension is combined with dyslipidemia, LDL is oxidized to oxLDL, which invades and deposits by damaging LOX-1 on the surface of vascular endothelial cell membrane [35], inhibits eNOS, reduces NO production, damages vasomotor function, increases blood pressure and activates RAAS, and aggravates myocardial fibrosis [36,37]. Hypertension itself is a complex disease driven by inflammation, oxidative stress and endothelial dysfunction, and dyslipidemia accelerates this process by stimulating inflammation and oxidative stress [38]. On this basis, LDL oxidative modification can further inhibit HDL function, such as reducing apoA-I activity and weakening cholesterol reverse transport efficiency [39,40]. HDL can inhibit LDL oxidation, and the two form a dynamic balance in the metabolic network [40]. However, it is worth noting that the vascular endothelial dysfunction, due to its complex pathological mechanism, has become a major challenge in clinical intervention. A thorough understanding of its pathogenesis and the development of targeted therapeutic drugs are of great scientific value for breaking through the bottlenecks in the prevention and treatment of hypertensive conditions. After injury in L-NNA-induced hypertensive rats, HE and Masson staining revealed thickened aortic walls, increased adventitial fibrosis, disordered extracellular matrix, fragmented elastic fibers, and enhanced collagen deposition in the Model group, all of which were markedly ameliorated by Edu treatment. Oil Red O staining demonstrated no significant lipid deposition in the thoracic aorta across all groups. Proteomic results of the thoracic aorta vessels analyzed differentially expressed protein-related KEGG pathways from multiple dimensions. The findings demonstrated that the co-regulated bubble map between the Model and Control groups showed protein enrichment in pathways including fatty acid degradation, the TCA cycle, pyruvate metabolism, and oxidative phosphorylation. These pathways are of significant importance for lipid metabolism and energy metabolism, and are closely associated with fatty acid elongation and myocardial contraction. In the comparison between the Edu and Model groups, significant intersections were observed in the TCA cycle, fatty acid degradation, oxidative phosphorylation, and fatty acid elongation pathways. Notably, in the fatty acid degradation pathway, the proteins up-regulated by Edu almost entirely corresponded to those down-regulated in the Control group, and their number was significantly greater than that in the SNP group.

The lipid test results in this study indicated that the L-NNA-induced hypertensive model rats mainly showed a decrease in HDL. After Edu treatment, compared with the Model group, LDL significantly decreased, suggesting that Edu improved the abnormal lipid metabolism under the state of hypertension. To clarify the protective mechanism of Edu in hypertensive rats, we conducted a serum metabolomics study using UPLC-QTOF-MS/MS technology to characterize the changes in the metabolic profile. The downstream small molecule differential metabolites generated mainly related to the following metabolic pathways: bile acid metabolism, energy metabolism, and lipid metabolism. Ursodeoxycholic acid (UDCA) is a naturally occurring secondary bile acid. In vivo, it conjugates with taurine to form Tauroursodeoxycholic acid (TUDCA) [41]. These compounds are key components of bile acid metabolism [42]; their conjugation with taurine enhances solubility in the bloodstream and efficiency of intestinal transport [43]. Within bile acid metabolism, TUDCA participates in the enterohepatic circulation, undergoing excretion and reabsorption via the taurine/glycine conjugation pathway [43,44]. Simultaneously, TUDCA modulates nuclear/membrane receptors such as the farnesoid X receptor (FXR) and G-protein-coupled bile acid receptor (TGR5) [45], thereby influencing bile acid synthesis enzymes [45], bile acid transporters, and consequently regulating bile acid synthesis, transport, and transporter activity to maintain bile acid pool homeostasis [46]. In the pathogenesis and progression of hypertension, bile acid metabolism dysregulation is closely associated with endothelial dysfunction [47], oxidative stress [48], inflammation [49], and abnormal activation of the renin–angiotensin–aldosteronerenin-angiotensin-aldosterone system (RAAS) [49]. Through FXR and TGR5 receptor-mediated pathways, TUDCA exerts antioxidant and anti-inflammatory, thereby exerting beneficial regulatory effects on blood pressure [50]. Consequently, TUDCA not only serves as a critical regulatory molecule in bile acid metabolism but also participates in the pathophysiological processes of hypertension via multiple pathway mechanisms, demonstrating potential metabolic-cardiovascular protective properties [51,52]. This study found that serum levels of UDCA and TUDCA were significantly elevated in L-NNA-induced hypertensive rats, whereas Edu treatment reversed this abnormal increase. This alteration may represent one of the underlying mechanisms through which Edu exerts its antihypertensive effects, reflecting its coordinated modulation of the bile acid–metabolism–vascular function axis.

Lipid metabolism alterations are a potential pathogenic mechanism of hypertension, as vascular dysfunction has been observed in both patients with dyslipidemia and hypertension [53]. In the present study, compared with the Control group, serum levels of 1-octadecanoyl-2-(9Z-octadecenoyl)-sn-glycero-3-phosphate (18:0–18:1(9Z)-PA) were significantly elevated in the Model group, whereas treatment with Edu reversed this abnormal increase. 18:0–18:1(9Z)-PA is a glycerophosphoric acid (PA) composed of the saturated fatty acid 18:0 and the monounsaturated fatty acid 18:1(9Z), and serves as a key intermediate in lipid metabolism [53]. It is involved in the synthesis of triacylglycerols and phospholipids, and functions as an important signaling molecule that regulates lipid synthesis and degradation, influencing lipogenesis and energy metabolism, thereby playing a central role in maintaining lipid homeostasis [54]. Excessive accumulation of 18:0–18:1(9Z)-PA can induce bile acid signaling dysregulation, promote vasoconstriction, sodium and water retention, and sympathetic overactivation, collectively increasing the risk of hypertension. Therefore, reducing the level of 18:0–18:1(9Z)-PA, improving lipid and bile acid metabolic disorders, and alleviating vasoconstriction and sodium-water retention may be the potential mechanism of Edu’s antihypertensive effect.

Fatty acid metabolism is closely related to the changes in blood pressure. Disordered fatty acid metabolism under hypertensive conditions represents a critical metabolic abnormality driving vascular endothelial injury and target organ damage. Elevated levels of free fatty acids (FFAs) exacerbate endothelial oxidative stress, reduce nitric oxide bioavailability, and impair endothelium-dependent vasodilation [55]. The accumulation of saturated fatty acids such as palmitic acid activates the NF-κB pathway, promoting smooth muscle proliferation and the release of inflammatory factors, thereby contributing to vascular remodeling and increased peripheral resistance [56]. Impaired fatty acid β-oxidation, characterized by diminished mitochondrial β-oxidation capacity, induces lipotoxicity, leading to mitochondrial dysfunction and cellular apoptosis [57]. Furthermore, an imbalance in the n-3/n-6 polyunsaturated fatty acid ratio, where thromboxane TXA_2_ derived from n-6 PUF promotes inflammation and vasoconstriction, coupled with attenuated anti-inflammatory and vasodilatory effects of n-3 PUFAs, further disrupts the regulation of vascular tension [58]. OXSM as a key enzyme involved in the initiation and elongation of fatty acid carbon chains, regulates the synthetic pathways and carbon chain structures of fatty acids, thereby influencing the composition and total levels of fatty acids in circulation [23]. MECR is a critical reductase in the mitochondrial β-oxidation process, whose function directly affects the efficiency of fatty acid degradation and the balance of energy metabolism—particularly in relation to the maintenance of mitochondrial function and the accumulation of lipotoxicity [24]. Therefore, the functional status of OXSM and MECR largely determines the “synthesis-degradation” balance of fatty acid metabolism, thereby participating in the regulation of the pathological processes associated with vascular and target organ damage in hypertension. Integrated analysis of differentially expressed proteins and metabolites in this study revealed that the expression levels of OXSM and MECR were significantly up-regulated in hypertensive rats. Notably, Edu treatment markedly reduced the abnormal expression of OXSM and MECR in these rats. These findings suggest that Edu may systematically modulate the balance of the fatty acid metabolic network by targeting the carbon chain initiation and elongation steps in fatty acid synthesis, as well as the key reductive reactions in mitochondrial β-oxidation. This regulatory effect may contribute to the improvement of endothelial dysfunction in the vasculature of hypertensive rats, the suppression of chronic inflammatory responses in the vascular wall, and the maintenance of vascular structural homeostasis.

## 5. Conclusions

This study confirmed that Edu has a protective effect on vascular endothelial injury induced by L-NNA in hypertensive rats through the detection of physiological and biochemical indicators and histopathological observation. Integrated metabolomics and proteomics analysis indicates that fatty acid metabolism is the key pathological axis of hypertension, and its homeostasis disorder directly causes damage to blood vessels and target organs. Edu significantly regulates fatty acid degradation in the thoracic aorta induced by L-NNA by targeting fatty acid β-oxidation, oxidative phosphorylation function, and TCA cycle activity. Consequently, it prevents the activation of fatty acid biosynthesis identified in serum metabolomics, ameliorates the paradoxical decrease in blood lipid levels, and counteracts the metabolic compensatory hyperactivity phenotype accompanied by reduced blood glucose. The up-regulation of OXSM and MECR levels disrupts the “synthesis-degradation” balance of fatty acids, while Edu improves endothelial dysfunction, inhibits vascular wall inflammation and maintains vascular structural homeostasis by restoring this balance. This may be the potential mechanism by which Edu exerts its antihypertensive effect. These findings help assess the therapeutic potential of Edu and provide a theoretical basis for optimizing the treatment strategies for hypertension.

## Data Availability

All data generated or analyzed during this study are included in this published article. Further inquiries can be directed to the corresponding authors.

## Supplementary Materials

The following supporting information can be downloaded at: https://www.mdpi.com/article/10.3390/cimb47120987/s1.
